# Two Funny Doctors

**Published:** 1882-11

**Authors:** 


### TWO FUNNY DOCTORS.

Two doctors have lately conspired to amuse the medical profession of the country with some jokes about prepared food, and their success has proved very gratifying. These jokers are Dr. Gaillard, editor of the *New York Medical Weekly*, and Dr. Ephraim Cutter, formerly of Boston. The latter wrote an article on foods, and Dr. Gaillard printed it. To the eye of the casual observer it seemed simply an honest and earnest effort to make known the composition of many of the cereal substances sold in New York. Cuts were given, in the form of large dotted discs. These discs purported to be microscopic fields, and the dots were said to indicate the substances discovered by the microscope in the several foods in question. Some of the dots were round, some oval, and some square. The round and oval dots were called "starch grains," the square ones were christened "gluten cells." The running commentary upon these pictures had an air of gravity and an assumption of science about it, well calculated to deceive. Dr. Gaillard aided his brother humorist by pretending to wax furious over the "frauds in prepared foods," which Dr. Cutter's pictures "exposed." He talked loftily about endangering the lives of infants with foods having a preponderance of round dots, and a paucity of square ones. He was awfully severe on the round dot fellows! Together, these two doctors succeeded in "selling" some very excellent people. Mr. Beach of the *Scientific American*, glanced at the stuff, imagined it to be sober, genuine science, and reprinted it in his "Supplement!" How Gaillard and Cutter—and the miller who "put up the job" in return for a disc full of square dots—chuckled and grinned! Dr. Jacoby squinted at the pictures, and, being a trifle myopic, failed to discover their impossible character as microscopic enlargements; he swallowed the jokes of the brace of humorists as scientific gospel, and embodied them in a paper on Infant Feeding! Dr. George B. Fowler mildly protested against the conclusions of the funny twins. Prof. Jos. G. Richardson, of Philadelphia, charged them with the perpetration of "a serious microscopic blunder." Prof. Thurston, of the Stevens Institute of Technology, was displeased because the flours of the Health Food Company of New York, which he and his family believed in—having derived great benefit from their use—did not have as good a picture, with as many square dots in it, as some of the other flours had. So he got Prof. Albert R. Leeds, Public Analyist for the State of New Jersey, and Professor of Chemistry in the Stevens Institute, to subject to chemical analysis several of the flours and foods which Cutter said he had examined. The result of

Prof. Leeds' honest chemical work opened people's eyes. He showed that no microscope could determine the relative proportions of gluten and starch in a given flour. This made it tolerably clear that Dr. Cutter's pictures did not represent flour at all. When, in continuation of his labors, Professor Leeds found that the flours of the Health Food Company were the best made in America, and that the flour of the "Franklin Mills"—which Cutter had earnestly commended as "a blessing to mankind"—was only a cheap mixture of finely ground bran and inferior flour—the joke was out! Where the jolly jokers got the dotted discs, was now the problem; but it was presently discovered that they were merely representations of the targets used in the International Rifle Match of 1877! The square dots represented the holes made by the bullets of the Irish team, which employed the famous square-cored rifle, throwing a square bullet, while the round dots were made by the round bullets of the American team. The cuts were bought at an old junk-shop, and rough handling had battered many of the round dots into ovals. In order to show a great many "gluten cells" for the "Franklin Mills" flour, the target of Captain McGinnis, the champion shot of the Irish team, was cunningly assigned to it, while, to make it appear that other flours contained nothing but starch, and not much of that, the targets dotted with the lowest scores of the poorest shots of the American team were assigned to them. Then the amusement of the physicians of Boston when Cutter's article was first shown to them, was understood. They all know him of old, and hold his science and his humor alike at their true value, and they feel certain, now that a fellow humorist of kindred genius has united with him for the entertainment of the profession, that its literature will bubble and sparkle with the quaint conceits of this amusing twinity, whose names are now and forever indissolubly associated, by the one touch of nature which makes the whole medical world grin!

Dr. Cutter's humor is irrepressible. He will have his little joke, no matter who suffers. On April 8th. the *New York Medical Record* printed an article from his pen on "Suspicious Organisms in Croton Water," with target accompaniment. This disc, said to represent an enlarged drop of Croton "taken from a bath-tub faucet," contained about a bushel of fish, flesh, fowl and crustacea. It is in reality a rich broth, taken from his bath tub, as he intimates, and is unquestionably a post-bath sample. On no other hypothesis can the presence in his picture of the very admirable specimen of the *pediculus humanus*, shown at L, and mistakenly characterized as *plagiophrys*, be accounted for.

